# Public Health Risk of *Campylobacter* spp. Isolated from Slaughterhouse and Retail Poultry Meat: Prevalence and Antimicrobial Resistance Profiles

**DOI:** 10.3390/pathogens14040316

**Published:** 2025-03-26

**Authors:** Sebastian Alexandru Popa, Viorel Herman, Emil Tîrziu, Adriana Morar, Alexandra Ban-Cucerzan, Mirela Imre, Răzvan-Tudor Pătrînjan, Kálmán Imre

**Affiliations:** 1 Faculty of Veterinary Medicine, University of Life Sciences “King Mihai I” from Timisoara, 300645 Timisoara, Romania; viorel.herman@fmvt.ro (V.H.); emiltirziu@usvt.ro (E.T.); adrianamorar@usvt.ro (A.M.); alexandracucerzan@usvt.ro (A.B.-C.); mirela.imre@usvt.ro (M.I.); razvan.patrinjan.fmv@usvt.ro (R.-T.P.); 2 Research Institute for Biosecurity and Bioengineering, University of Life Sciences ‘’King Mihai I” from Timisoara, 300645 Timisoara, Romania

**Keywords:** *Campylobacter* spp., microbiology, antimicrobial resistance (AMR), poultry, slaughterhouse, retail

## Abstract

*Campylobacter* spp. represents one of the most frequently incriminated pathogens in the evolution of foodborne gastroenteritis in humans worldwide. Alongside *Salmonella* spp., *Yersinia* spp., *Escherichia coli*, and *Listeria monocytogenes,* these pathogens represent a principal threat to public health because they are vehiculated to humans via food products and many of them have developed alarming resistance to different classes of antimicrobials. Thus, the present study aimed to provide scientifically relevant data on the public health risk represented by *Campylobacter* spp., contamination of chicken carcasses at the slaughterhouse and retail levels, and the antimicrobial resistance of the isolated strains. A total of 130 samples collected from slaughterhouses (n = 40) and retail stores (n = 90) were analyzed using standardized microbiological methods (ISO 10272-1:2017). Of these, the overall prevalence of *Campylobacter* spp. was 27.7%, with a prevalence at the slaughterhouse level of 32.5% and at the retail level of 25.5%. Following antimicrobial resistance profile determinations using the Kirby–Bauer disc diffusion assay, the isolated strains showed resistance to the following antimicrobials in descending order: ciprofloxacin (41.6%), tetracycline (25.0%), chloramphenicol (16.6%), gentamicin (11.1%), ertapenem (5.6%), and erythromycin (2.8%). The study results confirm that chicken meat may pose a threat to public health and, moreover, that due to the widespread use of antimicrobials, a large number of strains have developed antimicrobial resistance, leading to difficulties in the treatment of various foodborne diseases.

## 1. Introduction

Poultry meat has become one of the most consumed sources of animal protein worldwide due to its affordability, relatively low environmental impact, and high nutritional value [1]. According to the Food and Agriculture Organization (FAO), global poultry meat production reached almost 150 million tons in 2024, with significant contributions from the United States, Brazil, China, and the European Union [2]. Europe is a major player in the poultry industry, with over 15 million tons produced annually by leading countries such as Poland, France, or Spain [3]. The nutritional value of poultry meat is supported by its lower saturated fat content and higher polyunsaturated fatty acids, which contribute to its perceived role in promoting cardiovascular health [4,5]. Additionally, poultry meat serves as a valuable source of high-quality protein, essential vitamins—especially B3 and B6—and key minerals such as phosphorus and selenium, which collectively contribute to vital physiological functions, including energy metabolism, cognitive function, bone health, and immune system regulation [6,7]. Numerous studies have associated poultry consumption with positive health outcomes, including weight management and reduced risks of chronic diseases such as type-2 diabetes and hypertension.

Despite the economic and nutritional benefits of chicken meat, its production and consumption are linked to foodborne infections, *Campylobacter* spp. being the most common bacterial pathogen associated with poultry [8]. Additionally, poultry meat and its derived products can serve as significant reservoirs for other foodborne pathogens, posing substantial risks to public health. In addition to *Campylobacter*, other major pathogens such as *Salmonella* spp. [9], *Escherichia coli* (*E. coli*) [10], and *Listeria monocytogenes* (*L. monocytogenes*) [11] are frequently associated with poultry products, contributing to severe foodborne infections. *Salmonella* remains a predominant cause of salmonellosis outbreaks, while *L. monocytogenes* poses a serious threat due to its ability to survive and multiply under refrigeration conditions [12,13,14].

The epidemiology and the possibility of contamination, followed by the development of various food-borne pathologies by humans, caused by *Campylobacter* spp., are complex, with animals (both domestic and wild), water, the environment, and food being reported in many cases as sources of infection [15]. Contamination occurs most frequently at the slaughterhouse level. The results of several previously conducted investigations [16,17,18] demonstrated that slaughterhouses are regarded as an excellent level to mitigate the microbiological risk and prevent the transmission of bacterial agents with public health significance.

*Campylobacter* spp. can cause acute enteritis, post-infectious complications, and erratic, localized infections in humans. Symptoms may appear two to five days after infection and include acute abdominal pain, cramping diarrhea, often with fever and vomiting. The disease progresses over five to seven days [19,20,21].

The misuse of antimicrobials in poultry farming has significantly contributed to the emergence of antimicrobial resistance (AMR), posing a major threat to human health. The most alarming consequence of AMR in poultry production is the development of multidrug-resistant (MDR) bacteria, which can render critical antibiotics ineffective, making infections increasingly difficult, or even impossible, to treat [22]. These resistant pathogens can spread through contaminated food, direct contact, or environmental pathways, leading to severe health complications in humans. The World Health Organization recognizes AMR as one of the greatest global health threats, emphasizing the urgent need for stricter regulations, improved surveillance, and alternative disease control strategies to mitigate its impact [23,24].

Addressing these challenges is crucial to ensuring that poultry meat remains a viable, safe, and sustainable food source in the future.

Given the global prominence of poultry meat as a primary protein source, the continuous investigation into its production, safety, and public health implications remains imperative. In particular, the prevalence of zoonotic pathogens, such as *Campylobacter* spp., within retail poultry products has garnered significant attention due to their association with foodborne illness and their considerable public health burden [25]. The present study aims to determine the prevalence and antimicrobial resistance of *Campylobacter* spp. at the slaughterhouse level and in retail poultry products. Furthermore, the research will discuss the broader implications of *Campylobacter* spp. contamination in relation to public health policy, AMR, and the future sustainability of poultry production systems.

## 2. Materials and Methods

### 2.1. Sample Collection

The study was conducted from January 2024 to January 2025. During this period, a total of 130 samples represented by chicken meat (skin, fillets, backs, necks) were analyzed for the detection of *Campylobacter* spp. Raw chicken samples (never frozen) and neck skin samples were randomly procured and collected twice a month from retail units (n = 90) and slaughterhouses (n = 40) located in southern, western, and central counties of Romania.

All the samples (n = 130) were collected in sterile bags and transported in isothermal containers under refrigeration conditions (2–4 °C) to the Laboratory of Microbiology Risk Surveillance at the Faculty of Veterinary Medicine, Timișoara, and processed within 24 h after purchase.

### 2.2. Isolation and Identification of Campylobacter *spp.*

*Campylobacter* spp. isolates were detected and identified through direct plating in accordance with the ISO 10272-1:2017 standard [26].

The procedure was used for the detection of *Campylobacter* spp. through enrichment in the samples containing a small amount of microorganisms and low background microflora, or in instances of stressed campylobacteria, such as in cooked or frozen goods.

Firstly, a 1:10 dilution was prepared by combining 10 g of a sample with 90 mL of the enrichment medium (Bolton broth). The resulting mixture was then homogenized using a stomacher for 60 s to ensure uniformity. The initial suspension was subjected to incubation under controlled microaerobic conditions, initially at 37 °C for 4 to 6 h to facilitate initial microbial adaptation, followed by a prolonged incubation phase at 41.5 °C for 44 ± 4 h to optimize the growth and recovery of targeted microorganisms.

From the culture obtained in the enrichment medium, a 10 µL loopful of the suspension was aseptically streaked onto two selective agar media represented by the charcoal cefoperazone deoxycholate agar (mCCDA; Oxoid Ltd., Basingstoke, UK) and Butzler agar (Oxoid Ltd., Basingstoke, UK). These media were chosen for their specificity in isolating and recovering campylobacteria under selective growth conditions.

**Figure 1:**
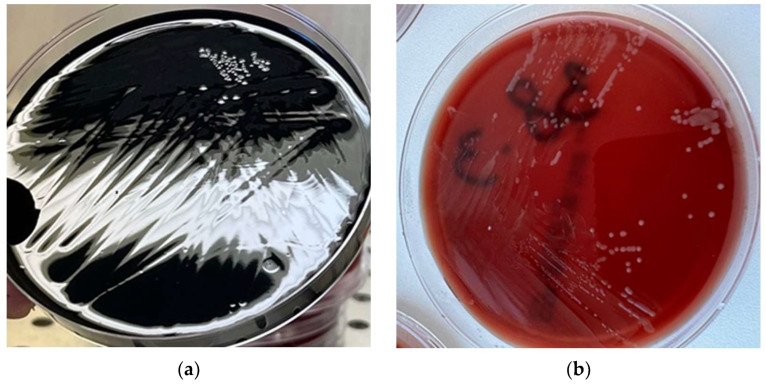
The appearance of *Campylobacter* spp. on (**a**) mCCDA and (**b**) Butzler selective agar.

The inoculated plates were incubated at 41.5 °C for 44 h under controlled microaerobic conditions (5% O_2_, 10% CO_2_, 85% N_2_) using anaerobic jars equipped with microaerobic gas-generating bags (Thermo Scientific™, Waltham, MA, USA). Following incubation, at least one specific colony displaying typical morphological characteristics of presumptive *Campylobacter* spp. as shown in Figure 1 (greyish with a metallic sheen, moist, flat, with a honey appearance—tendency to spread—on mCCDA (a); greyish with a metallic sheen or brown to orange in color—on Butzler agar (b)) was carefully selected and subcultured onto non-selective Columbia blood agar plates (Oxoid Ltd., Basingstoke, UK) to increase the recovery of pure isolates. The subcultured plates were then incubated under the same microaerobic conditions at 41.5 °C for an additional 44 h to ensure the development of isolated pure colonies.

After the completion of the incubation period, the freshly grown pure colonies were subjected to evaluation, including the assessment of their cellular morphology (seagull, spiral-shaped, S-shaped, or curved) using Gram staining under microscopy. Additionally, the isolates were identified using biochemical methods, including the catalase test, the oxidase test, and the DrySpot agglutination test. All the isolates exhibited positive catalase and oxidase activity, confirming their microaerobic metabolism characteristics. Additionally, the DrySpot agglutination test yielded positive results, further confirming the identification of *Campylobacter* spp. Additionally, the absence of aerobic growth capacity at 25 °C was tested, as described in the ISO 10272-1:2017 standard. *Campylobacter jejuni* ATCC 33291 and *Campylobacter coli* ATCC 43478 were used as positive control strains.

All of the isolated strains identified using microbiological methods were subjected to molecular analysis. The extraction of the genomic DNA was carried out using an ISOLATE II Genomic DNA Kit (BIOLINE^®^ UK Ltd., London, UK). The molecular confirmation of the isolated strains at the genus level was accomplished through a conventional polymerase chain reaction (PCR) targeting the 23S rRNA gene and using the 23SF forward (5′-TA-TACCGGTAAGGAGTGCTGGAG-3′) and 23SR reverse (5′-ATCAATTAACCTTCGAGCACCG-3′) specific primer set, and cycling conditions as previously described by Wang et al. [27]. The analysis and control of the resulting amplicons were carried out using horizontal electrophoresis in a submerged electrophoresis system in 1.5% agarose gel, with the addition of the fluorescent dye RedSafe™ (iNtRON Biotechnology, Inc., Gyeonggi-do, South Korea). As a marker, the 100 bp DNA ladder (BIOLINE^®^ UK Ltd., London, UK) was used in the first well of the gel. DNA fragments were visualized using a UV photodocumentation system (UVP^®^).

### 2.3. Antimicrobial Susceptibility Tests

A confirmed and purely isolated strain from each *Campylobacter*-positive sample was selected for antimicrobial susceptibility testing. The susceptibility assessment was conducted using the Kirby–Bauer disk diffusion method on Mueller–Hinton agar (Oxoid Ltd., Basingstoke, UK) supplemented with 5% defibrinated horse blood, following the guidelines of the Clinical and Laboratory Standards Institute. Mueller–Hinton agar plates were inoculated with a 0.5 McFarland standard suspension and incubated at 42 °C for 48 h under microaerophilic conditions. After incubation, the diameters of the inhibition zones were measured, analyzed, and classified as susceptible (S), intermediate (I), and resistant (R) according to the interpretive criteria established by the European Committee on Antimicrobial Susceptibility Testing (EUCAST) for ertapenem, gentamicin, and chloramphenicol and by the Clinical and Laboratory Standards Institute (CLSI) for *Campylobacter* spp. and Enterobacteriaceae for erythromycin, ciprofloxacin, and tetracycline [28,29,30]. The antimicrobial tested classes included fluoroquinolones (ciprofloxacin—5 µg/disc), amphenicols (chloramphenicol—30 µg/disc), macrolides (erythromycin—15 µg/disc), carbapenems (ertapenem—5 µg/disc), aminoglycosides (gentamicin—10 µg/disc), tetracyclines (tetracycline—30 µg/disc), monitored within the National Program for Antimicrobial Resistance of *Campylobacter* spp. strains and for EFSA reporting on antimicrobial resistance data under Directive 2003/99/EC and Commission Implementing Decision (EU) 2020/1729 [31].

### 2.4. Statystical Analyses

Statistical analyses were performed using GraphPad version 10.0.2 (Boston, MA, USA). The prevalence of *Campylobacter* spp. and antimicrobial resistance rates were calculated as percentages with 95% confidence intervals (CI). Comparisons between *Campylobacter* prevalence in different sample sources (retail vs. slaughterhouse) were analyzed using the chi-squared test (χ^2^ test). Similarly, differences in antimicrobial resistance profiles among the tested antibiotics were evaluated using a chi-squared test for categorical data to determine statistical significance. A *p*-value of <0.05 was considered statistically significant. The statistical results were interpreted based on the obtained χ^2^ values and the corresponding *p*-values, where *p* < 0.05 indicated a significant difference and *p* ≥ 0.05 suggested no significant difference between the analyzed groups.

## 3. Results

### 3.1. Campylobacter *spp.* Prevalence

A total of 130 poultry meat samples obtained from the retail and slaughterhouse levels were analyzed to determine the prevalence of *Campylobacter* spp. Out of them, 36 samples yielded positive results, leading to an overall prevalence of 27.7%. The results are summarized in Table 1.

Molecular confirmation of the isolated *Campylobacter* strains based on the PCR amplification of the partial sequence of the 23S rRNA gene, was successfully achieved for all (n = 36) the isolates. The samples obtained from slaughterhouses, particularly neck skin, indicated a contamination rate of 32.5% (13/40; 95% CI, 20.1–48.0), which was similar to the prevalence found in the retail neck samples. The findings indicate that specific anatomical regions, including the neck and neck skin, exhibit heightened vulnerability to *Campylobacter* infection, presumably due to greater exposure to intestinal contents during processing. The chi-squared analysis comparing the prevalence of *Campylobacter* spp. between the slaughterhouse and retail level samples revealed no statistically significant difference (χ^2^ = 0.365, *p* = 0.546), suggesting that contamination levels remain relatively constant throughout the processing and distribution chain. This finding indicates that while some decontamination measures may be in place at slaughterhouses, they might not be sufficient to significantly reduce the *Campylobacter* presence by the time the meat reaches consumers. The similar prevalence rates at both levels emphasize the need for more effective interventions throughout the poultry supply chain, specifically in slaughtering and processing facilities where cross-contamination can occur.

Contamination rates varied among different sample types, influenced by the anatomical region and sample origin. The highest incidence in the retail meat items was observed in the neck samples, with 11 out of 30 (36.7%; 95% CI, 21.9–54.5) testing positive. *Campylobacter* spp. was identified in 7/30 (23.3%; 95% CI, 11.8–40.9) fillet samples and 5/30 (16.7%; 95% CI, 7.3–33.6) back samples.

### 3.2. Antimicrobial Susceptibility Test Results

The antimicrobial susceptibility profile of the isolated strains was also tested in the present study. The results are presented in Table 2.

A total of 36 *Campylobacter* isolates were evaluated against six antimicrobial drugs, demonstrating differing levels of susceptibility, intermediate resistance, and resistance. The findings underscore alarming resistance patterns, especially for fluoroquinolones (ciprofloxacin) and tetracycline, while other antimicrobials, such as erythromycin and ertapenem, maintained substantial efficacy. Ciprofloxacin, a fluoroquinolone frequently used for the treatment of campylobacteriosis in humans, showed a significant resistance rate of 41.6%, with 50.0% of the isolates exhibiting susceptibility and 8.4% indicating intermediate resistance. Chloramphenicol resistance was detected in 16.6% of the isolates, with no intermediate resistance, while the majority of the isolated strains (83.4%) remained susceptible. The relatively low resistance may be attributed to its limited use in veterinary medicine. Erythromycin showed the highest susceptibility rate (94.4%), with only 2.8% resistance, confirming its continued effectiveness as the primary treatment for human campylobacteriosis. The low resistance levels suggest minimal macrolide use in poultry production compared to other antibiotics. Gentamicin resistance was observed in 11.1% of the isolates, while 88.9% were susceptible. Though the resistance levels were relatively low, continued monitoring is necessary, as gentamicin is an important therapeutic option for severe infections.

A high resistance rate (25.0%) was observed for tetracycline, with 4/36 (11.1%) of the isolates exhibiting intermediate resistance. Given its frequent use in poultry farming, this finding underscores the need for stricter regulations on antibiotic use to prevent the further spread of tetracycline-resistant *Campylobacter* strains. Ertapenem exhibited 94.4% susceptibility, with 2 (5.6%) resistant isolates.

The chi-squared test for antimicrobial resistance demonstrated a highly significant difference among the resistance rates across the tested antibiotics (χ^2^ = 40.926, *p* < 0.001). This indicates that the *Campylobacter* isolates exhibited varying degrees of resistance, with certain antimicrobials being more affected than others. The statistical confirmation of the significant differences in the resistance rates underlines the growing risk of AMR evolution in *Campylobacter* spp., particularly in poultry-associated strains.

The antimicrobial resistance patterns of the *Campylobacter* spp. isolates were further analyzed to assess the multidrug resistance (MDR) profiles. The distribution of the isolates based on their resistance to multiple antimicrobial classes is presented in Table 3.

Six isolates exhibited resistance to two antimicrobial classes. Four isolates were resistant to ciprofloxacin and tetracycline, indicating fluoroquinolone and tetracycline resistance, which are commonly associated with *Campylobacter* spp. infections. Two isolates showed resistance to gentamicin and tetracycline, representing aminoglycoside and tetracycline resistance. Two isolates were resistant to ciprofloxacin, chloramphenicol, and tetracycline, indicating resistance to fluoroquinolones, amphenicols, and tetracyclines. One isolate was resistant to ciprofloxacin, chloramphenicol, tetracycline, and gentamicin, indicating fluoroquinolone, amphenicol, tetracycline, and aminoglycoside resistance. This MDR profile is concerning, as it significantly limits treatment options for *Campylobacter* infections in humans.

## 4. Discussion

To the best of the authors’ knowledge, unfortunately, in Romania, scientific investigations on *Campylobacter* spp. prevalence and antimicrobial resistance in poultry meat products are limited, with few research studies [32,33] addressing this critical public health issue.

Our study identified a *Campylobacter* prevalence of 25.6% at the retail level, which is approximately the same as the overall prevalence of 27.7%, but still significant from the food safety perspective. This outcome indicates that although poultry meat is subjected to certain decontamination processes prior to consumer distribution, these interventions may not completely eradicate *Campylobacter* contamination [34,35]. A study conducted in Transylvania, Romania, indicated a *Campylobacter* prevalence of 29.4% in retail poultry meat [33]. This number slightly exceeds our 25.5% retail prevalence, indicating that geographical variations in poultry production and distribution may affect contamination rates. The difference could also result from differences in sample varieties, testing procedures, or the implementation of more rigorous control and biosecurity measures within slaughterhouses.

The present study reported that the prevalence of *Campylobacter* spp. in retail poultry products aligns with previous research in other European Union countries, such as Italy, where Stella et al. [20] reported a 34.1% prevalence in retail poultry meat. Both findings underscore the substantial prevalence of *Campylobacter* spp. in poultry products; however, variations in sample types, processing conditions, and food safety protocols may explain the observed variability. The current research revealed that *Campylobacter* prevalence was higher in the retail neck samples (36.7%) and the slaughterhouse neck skin samples (32.5%), whereas lower prevalence rates were identified in the fillets (23.3%) and the backs (16.7%). The Italian investigation revealed a comparable trend, with sectioned meats (those with skin) demonstrating the highest contamination rates (86.8%), whereas minced meat dishes showed markedly lower prevalence levels (22.4%). The increased contamination in skin-on samples suggests that *Campylobacter* predominantly inhabits poultry skin as a result of fecal contamination during slaughter and processing [36].

Considering other European studies assessing the presence of *Campylobacter* spp. in slaughterhouses or at the retail level, higher prevalence values were reported in Croatia (41.0%) [37], Estonia (63.3%) [38], Poland (64%) [39], France (76%) [40], Greece (70.4%) [41], and Ireland (84.3%) [42]. As demonstrated in the present study and the investigations mentioned above, in terms of comparison, the possibility of contamination of poultry carcasses with *Campylobacter* at the time of their delivery to the retail level is high. Due to this fact, presently, poultry processing companies are actively seeking a chemical-free decontamination method as an alternative. In this regard, the dielectric barrier discharge cold plasma technology can constitute a valuable tool capable of markedly reducing the bacterial load of contaminated carcasses [43].

The prevalence of *Campylobacter* spp. in poultry meat underscores the persistent risk of foodborne transmission, while the antibiotic resistance patterns of the isolated strains represent a significant concern.

Regarding AMR, the detected resistance rate of the isolated strains to ciprofloxacin (41.6%) was lower than the 79.1% reported in Italy [44], but higher than the 24.4% resistance rate identified in North Carolina [45]. The persistence of fluoroquinolone resistance even in antibiotic-free poultry farming, as shown by Poudel et al. [46] with a 49.2% resistance rate, suggests that resistance genes are already well-established in *Campylobacter* populations, likely due to past antibiotic usage in poultry production. Tetracycline resistance (25.0%) was significantly lower than the 90.7% resistance reported in Italy [44] and 64.3% in North Carolina [45]. However, this rate closely aligns with the 23.7% resistance rate reported in antibiotic-free poultry [46]. Erythromycin, a macrolide considered the first-line treatment for human campylobacteriosis, exhibited a resistance rate of only 2.8% in our study, which is notably lower than the 34.8% resistance reported in North Carolina [45] and the high levels observed in *C. coli* isolates from South Korea [47]. This finding reinforces the continued efficacy of macrolides in treating *Campylobacter* infections in Romania.

The identified resistance of the isolated strains to gentamicin and chloramphenicol in the present study was lower than the resistance reported in Italy [44] and North Carolina [45]. Even though chloramphenicol is no longer commonly used in poultry farming, resistance genes can persist in bacterial populations due to historical antibiotic use or co-selection with other antibiotics [48,49].

Tîrziu et al. [33] published research in 2020, in which they evaluated the antimicrobial resistance profile of *Campylobacter* strains isolated from poultry meat in Transylvania, Romania. The rates of resistance to ciprofloxacin and tetracycline in the present study were lower compared to the resistance rates obtained in the previous study. However, in the present study, a 11.1% resistance was identified for gentamicin, which is higher than the total susceptibility to this antimicrobial previously identified. Low resistance rates to erythromycin were identified in both studies [33].

An important result of the present study is represented by the isolates’ resistance to ertapenem. Even if the resistant rate was very low (5.6%), it is still alarming. Given that carbapenems are not commonly used in veterinary medicine, this resistance may indicate the presence of acquired mechanisms, such as carbapenem production or efflux pump overexpression, which require further investigation [50,51]. The emergence of ertapenem-resistant *Campylobacter* raises significant public health concerns, as carbapenems are considered last-resort antibiotics for severe bacterial infections in humans.

The detection of antimicrobial-resistant *Campylobacter* strains, including resistance to ciprofloxacin, tetracycline, and chloramphenicol, poses a significant challenge to both human and animal health. Recent findings from a study on *Campylobacter jejuni* isolates in Romania underscore the alarming prevalence of resistance mechanisms, including mutations in the gyrA gene conferring fluoroquinolone resistance and point mutations in the 23S rRNA gene associated with macrolide resistance. These resistance patterns not only limit therapeutic options for human infections, but also indicate a broader issue of antibiotic misuse in animal husbandry, necessitating stringent surveillance and control measures to mitigate the spread of multidrug-resistant strains [52]. Additionally, the emergence of resistance, even in antimicrobials with restricted use in food-producing animals, highlights the potential for cross-resistance and environmental persistence. A “One Health” strategy is essential to reduce unnecessary antibiotic use in animal agriculture and prevent the further spread of AMR through the food supply. Using the “One Health” framework in poultry farming requires a multifaceted approach that includes better biosecurity measures, smarter use of antibiotics, and stricter rules for food safety. By stepping up surveillance in slaughterhouses, stores, and hospitals, resistant strains can be found early on, protecting both humans and animals in the long run.

## 5. Conclusions

The isolation rate of campylobacters (27.7%) in the two links of the production chain indicates that this foodborne pathogen continues to pose a threat to public health in Romania. Improved hygiene measures should be enforced, in particular on the processing line at slaughterhouses. High resistance rates to ciprofloxacin and tetracycline suggest frequent use of these antimicrobials at poultry farms. While the overall resistance to erythromycin and ertapenem was low in our study, their detection in *Campylobacter* isolates remains alarming. Erythromycin is the first-line treatment for human campylobacteriosis, and any emergence of resistance threatens its clinical efficacy. Similarly, the identification of ertapenem-resistant strains is particularly concerning, as carbapenems are the last-resort antibiotics for severe bacterial infections. Antimicrobial resistance of *Campylobacter* isolates highlights a potential threat to the efficacy of therapeutic protocols and underlines the urgent need for continuous surveillance and responsible use of antibiotics in veterinary and human medicine.

Even if the results are promising and offer important insights about the risk of *Campylobacter* spp. to public health in Romania, further studies involving species identification, a larger amount of samples from all over Romania, together with molecular characterization of the isolated strains, especially on virulence and AMR genes, should be performed.

## Figures and Tables

**Table 1:** The prevalence of *Campylobacter* spp. in poultry meat samples from the retail and slaughterhouse levels.

Origin	Type of Samples	Number of Samples	Number of Positive Samples	Prevalence, % [95% CI]
**Slaughterhouse**	Neck skin	40	13	32.5% [20.1–48.0]
**Retail**	Fillets	30	7	23.3% [11.8–40.9]
	Backs	30	5	16.7% [7.3–33.6]
	Necks	30	11	36.7% [21.9–54.5]
**Total**	**-**	**130**	**36**	**27.7%**

**Table 2:** Antimicrobial resistance profiles of the *Campylobacter* spp. isolates from poultry meat.

Antimicrobials	S (%)	I (%)	R (%)
Ciprofloxacin	18 (50.0%)	3 (8.4%)	15 (41.6%)
Chloramphenicol	30 (83.4%)	0 (0.0%)	6 (16.6%)
Erythromycin	34 (94.4%)	1 (2.8%)	1 (2.8%)
Ertapenem	34 (94.4%)	0 (0.0%)	2 (5.6%)
Gentamicin	32 (88.9%)	0 (0.0%)	4 (11.1%)
Tetracycline	23 (63.9%)	4 (11.1%)	9 (25.0%)

Legend: S—susceptible; I—intermediate; R—resistant.

**Table 3:** Antimicrobial resistance phenotypes of the tested *Campylobacter* spp. strains.

Resistance Category	Antimicrobial Resistance Profile	Number of Isolates	Classes with Resistance
Against two	CIP + TET	4	fluoroquinolones, tetracyclines
	GEN + TET	2	aminoglycosides, tetracyclines
Against three	CIP + CHL + TET	2	fluoroquinolones, amphenicols, tetracyclines
Against four	CIP + CHL + TET + GEN	1	fluoroquinolones, amphenicols, tetracyclines, aminoglycosides

Legend: CIP—ciprofloxacin; TET—tetracycline; GEN—gentamicin; CHL—chloramphenicol.

## Data Availability

Data are contained within the article.

## References

[1] Gržinić G., Piotrowicz-Cieślak A., Klimkowicz-Pawlas A., Górny R.L., Ławniczek-Wałczyk A., Piechowicz L., Olkowska E., Potrykus M., Tankiewicz M., Krupka M. (2023). Intensive Poultry Farming: A Review of the Impact on the Environment and Human Health. Sci. Total Environ..

[2] Food and Agriculture Organization of the United Nations (FAO) Meat Market Review: Emerging Trends and Outlook. https://openknowledge.fao.org/items/9e01dd92-9217-47e8-8134-90a8955e30c0.

[3] European Commission (2021). EU Agricultural Outlook for Markets, Income and Environment 2021–2031.

[4] Wahyono N.D., Utami M.M.D. (2018). A Review of the Poultry Meat Production Industry for Food Safety in Indonesia. J. Phys. Conf. Ser..

[5] Connolly G., Campbell W.W. (2023). Poultry Consumption and Human Cardiometabolic Health-Related Outcomes: A Narrative Review. Nutrients.

[6] Fletcher D.L. (2002). Poultry meat quality. World’s Poult. Sci. J..

[7] Petracci M., Cavani C. (2012). Muscle growth and poultry meat quality issues. Nutrients.

[8] European Food Safety Authority (EFSA), European Centre for Disease Prevention and Control (ECDC) (2024). The European Union One Health 2023 Zoonoses report. EFSA J..

[9] Antunes P., Mourão J., Campos J., Peixe L. (2016). Salmonellosis: The role of poultry meat. Clin. Microbiol. Infect..

[10] Musa L., Proietti P.C., Marenzoni M.L., Stefanetti V., Kika T.S., Blasi F., Magistrali C.F., Toppi V., Ranucci D., Branciari R. (2021). Susceptibility of Commensal *E. coli* Isolated from Conventional, Antibiotic-Free, and Organic Meat Chickens on Farms and at Slaughter toward Antimicrobials with Public Health Relevance. Antibiotics.

[11] Jamshidi A., Zeinali T. (2019). Significance and Characteristics of *Listeria monocytogenes* in Poultry Products. Int. J. Food Sci..

[12] Diarrassouba F., Diarra M.S., Bach S., Delaquis P., Pritchard J., Topp E., Skura B.J. (2007). Antibiotic resistance and virulence genes in commensal *Escherichia coli* and *Salmonella* isolates from commercial broiler chicken farms. J. Food Prot..

[13] Popa S.A., Morar A., Ban-Cucerzan A., Imre K. (2022). Last decade mini-review of the scientific progresses in the monitoring of the occurrence and antimicrobial susceptibility profile of poultry origin *Campylobacter* spp. within the European Union countries. Rev. Rom. Med. Vet..

[14] Ștefan G., Predescu C., Bărăităreanu S. (2024). Evaluation of growth potential of *Listeria monocytogenes* in meat products ready to eat. Rev. Rom. Med. Vet..

[15] Wagenaar J.A., Newell D.G., Kalupahana R.S., Mughini-Gras L. (2023). *Campylobacter*: Animal reservoirs, human infections, and options for control. Zoonoses: Infections Affecting Humans and Animals.

[16] Silva J., Leite D., Fernandes M., Mena C., Gibbs P.A., Teixeira P. (2011). *Campylobacter* spp. as a foodborne pathogen: A review. Front. Microbiol..

[17] Sala C., Morar A., Tîrziu E., Nichita I., Imre M., Imre K. (2016). Environmental occurrence and antibiotic susceptibility profile of *Listeria monocytogenes* at a slaughterhouse raw processing plant in Romania. J. Food Prot..

[18] Ciui S., Morar A., Tîrziu E., Herman V., Ban-Cucerzan A., Popa S.A., Morar D., Imre M., Olariu-Jurca A., Imre K. (2023). Causes of Post-Mortem Carcass and Organ Condemnations and Economic Loss Assessment in a Cattle Slaughterhouse. Animals.

[19] Skarp C.P.A., Hännien M.L., Rautelin H.I.K. (2016). Campylobacteriosis: The role of poultry meat. Clin. Microbiol. Infect..

[20] Stella S., Soncini G., Ziino G., Panebianco A., Pedonese F., Nuvoloni R., Di Giannatale E., Colavita G., Alberghini L., Giaccone V. (2017). Prevalence and quantification of thermophilic *Campylobacter* spp. in Italian retail poultry meat: Analysis of influencing factors. Food Microbiol..

[21] Popa S.A., Morar A., Ban-Cucerzan A., Tîrziu E., Herman V., Imre M., Florea T., Morar D., Pătrânjan R.T., Imre K. (2024). First study on the frequency of isolation and phenotypic antimicrobial resistance profiles of pig- and cattle-origin *Campylobacter* strains in Romania. Vet. Res. Commun..

[22] Mak P.H., Rehman M.A., Kiarie E.G., Topp E., Diarra M.S. (2022). Production systems and important antimicrobial resistant-pathogenic bacteria in poultry: A review. J. Anim. Sci. Biotechnol..

[23] World Health Organization (2021). Global Antimicrobial Resistance and Use Surveillance System (GLASS) Report 2021.

[24] Barata R., Saavedra M.J., Almeida G. (2024). A Decade of Antimicrobial Resistance in Human and Animal *Campylobacter* spp. Isolates. Antibiotics.

[25] Myintzaw P., Jaiswal A.K., Jaiswal S. (2022). A review on Campylobacteriosis associated with poultry meat consumption. Food Rev. Int..

[26] (2017). Microbiology of the Food Chain–Horizontal Method for Detection and Enumeration of *Campylobacter* spp.–Part 1: Detection Method.

[27] Wang G., Clark C.G., Taylor T.M., Pucknell C., Barton C., Price L., Woodward D.L., Rodgers F.G. (2002). Colony multiplex PCR assay for identification and differentiation of *Campylobacter jejuni*, *C. coli*, *C. lari*, *C. upsaliensis*, and *C. fetus* subsp. fetus. J. Clin. Microbiol..

[28] Clinical and Laboratory Standards Institute (CLSI) (2015). Methods for Antimicrobial Dilution and Disk Susceptibility Testing of Infrequently Isolated or Fastidious Bacteria. CLSI Document M45.

[29] Karikari A.B., Obiri-Danso K., Frimpong E.H., Krogfelt K.A. (2017). Antibiotic resistance of *Campylobacter* recovered from faeces and carcasses of healthy livestock. BioMed Res. Int..

[30] The European Committee on Antimicrobial Susceptibility Testing Breakpoint Tables for Interpretation of MICs and Zone Diameters. Version 14.0, 2024. http://www.eucast.org.

[31] Amore G., Beloeil P.-A., Garcia Fierro R., Rizzi V., Stoicescu A.-V. (2025). Manual for reporting 2024 antimicrobial resistance data under Directive 2003/99/EC and Commission Implementing Decision (EU) 2020/1729. EFSA Support. Publ..

[32] Dan S., Tăbăran A., Mihaiu L., Mihaiu M. (2015). Antibiotic susceptibility and prevalence of foodborne pathogens in poultry meat in Romania. J. Infect. Dev. Ctries..

[33] Tîrziu E., Bărbălan G., Morar A., Herman V., Cristina R.T., Imre K. (2020). Occurrence and antimicrobial susceptibility profile of *Salmonella* spp. in raw and ready-to-eat foods and *Campylobacter* spp. in retail raw chicken meat in Transylvania, Romania. Foodborne Pathog. Dis..

[34] Hue O., Le Bouquin S., Laisney M.-J., Allain V., Lalande F., Petetin I., Rouxel S., Quesne S., Gloaguen P.-Y., Picherot M. (2010). Prevalence of and risk factors for *Campylobacter* spp. contamination of broiler chicken carcasses at the slaughterhouse. Food Microbiol..

[35] Perez-Arnedo I., Gonzalez-Fandos E. (2019). Prevalence of *Campylobacter* spp. in Poultry in Three Spanish Farms, A Slaughterhouse and A Further Processing Plant. Foods.

[36] Berrang M.E., Buhr R.J., Cason J.A., Dickens J.A. (2001). Broiler Carcass Contamination with *Campylobacter* from Feces during Defeathering. J. Food Prot..

[37] Kovačić A., Listeš I., Vučica C., Kozačinski L., Tripković I., Siško-Kraljević K. (2013). Distribution and genotypic characterization of *Campylobacter jejuni* isolated from poultry in Split and Dalmatia County, Croatia. Zoonoses Public Health.

[38] Mäesaar M., Kramarenko T., Meremäe K., Sõgel J., Lillenberg M., Häkkinen L., Ivanova M., Kovalenko K., Hörman A., Hänninen M. (2016). Antimicrobial Resistance Profiles of *Campylobacter* spp. Isolated from Broiler Chicken Meat of Estonian, Latvian and Lithuanian Origin at Estonian Retail Level and from Patients with Severe Enteric Infections in Estonia. Zoonoses Public Health.

[39] Szosland-Fałtyn A.N.N.A., Bartodziejska B., Krolasik J., Paziak-Domańska B.E.A.T.A., Korsak D., Chmiela M. (2018). The Prevalence of *Campylobacter* spp. in polish poultry meat. Pol. J. Microbiol..

[40] Guyard-Nicodème M., Rivoal K., Houard E., Rose V., Quesne S., Mourand G., Rouxel S., Kempf I., Guillier L., Gauchard F. (2015). Prevalence and characterization of *Campylobacter jejuni* from chicken meat sold in French retail outlets. Int. J. Food Microbiol..

[41] Natsos G., Mouttotou N.K., Magiorkinis E., Ioannidis A., Rodi-Burriel A., Chatzipanagiotou S., Koutoulis K.C. (2020). Prevalence of and risk factors for *Campylobacter* spp. colonization of broiler chicken flocks in Greece. Foodborne Pathog. Dis..

[42] Madden R.H., Moran L., Scates P., McBride J., Kelly C. (2011). Prevalence of *Campylobacter* and *Salmonella* in Raw Chicken on Retail Sale in the Republic of Ireland. J. Food Prot..

[43] Abdel-Naeem H.H., Ebaid E.M., Khalel K.H., Imre K., Morar A., Herman V., El-Nawawi F.A. (2022). Decontamination of chicken meat using dielectric barrier discharge cold plasma technology: The effect on microbial quality, physicochemical properties, topographical structure, and sensory attributes. LWT.

[44] Nobile C.G.A., Costantino R., Bianco A., Pileggi C., Pavia M. (2013). Prevalence and Pattern of Antibiotic Resistance of *Campylobacter* spp. in Poultry Meat in Southern Italy. Food Control.

[45] Hull D.M., Harrell E., van Vliet A.H.M., Correa M., Thakur S. (2021). Antimicrobial resistance and interspecies gene transfer in *Campylobacter coli* and *Campylobacter jejuni* isolated from food animals, poultry processing, and retail meat in North Carolina, 2018–2019. PLoS ONE.

[46] Poudel S., Li T., Chen S., Zhang X., Cheng W.-H., Sukumaran A.T., Kiess A.S., Zhang L. (2022). Prevalence, Antimicrobial Resistance, and Molecular Characterization of *Campylobacter* Isolated from Broilers and Broiler Meat Raised without Antibiotics. Microbiol. Spectr..

[47] Wei B., Cha S.Y., Yoon R.H., Kang M., Roh J.H., Seo H.S., Lee J.A., Jang H.K. (2016). Prevalence and antimicrobial resistance of *Campylobacter* spp. isolated from retail chicken and duck meat in South Korea. Food Control.

[48] Rivera-Mendoza D., Martínez-Flores I., Santamaría R.I., Lozano L., Bustamante V.H., Pérez-Morales D. (2020). Genomic Analysis Reveals the Genetic Determinants Associated with Antibiotic Resistance in the Zoonotic Pathogen *Campylobacter* spp. Distributed Globally. Front. Microbiol..

[49] Ma L., Konkel M.E., Lu X. (2021). Antimicrobial Resistance Gene Transfer from *Campylobacter jejuni* in Mono-and Dual-Species Biofilms. Appl. Environ. Microbiol..

[50] Yao H., Shen Z., Wang Y., Deng F., Liu D., Naren G., Dai L., Su C.-C., Wang B., Wang S. (2016). Emergence of a Potent Multidrug Efflux Pump Variant That Enhances *Campylobacter* Resistance to Multiple Antibiotics. mBio.

[51] Davin-Regli A., Pages J.-M., Ferrand A. (2021). Clinical Status of Efflux Resistance Mechanisms in Gram-Negative Bacteria. Antibiotics.

[52] Baltoiu M., Gradisteanu Pircalabioru G., Cristea D., Sorokin M., Dragomirescu C.C., Stoica I. (2024). Genetic Diversity, Virulence, and Antibiotic Resistance Determinants of *Campylobacter jejuni* Isolates in Romania. Pathogens.

